# Psychometric data concerning the Positive and Negative Affect Schedule – Direction (PANAS-D) in athletes in Arabic-Tunisian language supporting a 2-factor structure of its short modified version

**DOI:** 10.1016/j.dib.2017.02.019

**Published:** 2017-02-13

**Authors:** Sofiane Mnadla, Ali Elloumi, Jamel Hajji, Nicola L. Bragazzi

**Affiliations:** a Institute of Sport and Physical Education (ISSEP), The Department of Education Sciences, Faculty of Humanities and Social Sciences, University of Mannouba, Tunisia; b Department of Arts and Social Sciences, Sfax University, Tunisia; c Laboratoire Techniques et Enjeux du Corps, Université Paris Descartes, France; d Higher Institute of Sport and Physical Education of Gafsa, Gafsa University, Gafsa, Tunisia; e School of Public Health, Department of Health Sciences (DISSAL), University of Genoa, Via Antonio Pastore 1, Genoa 16132, Italy

**Keywords:** Semi-confirmatory factor analysis, Sport and exercise psychology

## Abstract

The Positive and Negative Affect Schedule – Direction (PANAS-D) questionnaire was translated from the French version developed by Nicolas and coworkers into Arabic-Tunisian language and administered to a sample of 519 athletes (mean age 19.43±3.78 years; 230 male, 229 female; 75 competing at international level, 287 at national level, 130 at regional level, and 27 at local level). A semi-confirmatory factor analysis was carried out in order to shed light on the factor structure of the questionnaire. Different models were tested, including the 1-factor, the 2-factor and the 3-factor structure models, and compared in terms of fitting indexes. Data support a 2-factor solution of the modified short version of the PANAS-D questionnaire.

**Specifications Table**

**Table:** 

Subject area	*Psychology*
More specific subject area	*Sport and exercise psychology*
Type of data	*Tables*
How data were acquired	*Administration of the questionnaire and analysis of data*
Data format	*Raw, analyzed*
Experimental factors	*Factor structure models, fitting indexes and factor loadings and multivariate analysis*
Experimental features	*Validation of the questionnaire through a semi-confirmatory factor analysis*
Data source location	*Tunisia*
Data accessibility	*Data are within this article*

**Value of the data**

• To the best of our knowledge, this is the first translation of the Positive and Negative Affect Schedule – Direction (PANAS-D) questionnaire in Arabic-Tunisian language.
• These data could be useful for Arabic researchers in that could be used for further investigation in the field of sport and exercise psychology, both for replicating our findings and for discovering new ones.
• These data could be useful for the scientific community in that could be used to shed light on the factor structure of the PANAS-D questionnaire.

## 1 Data

This paper contains psychometric data on the Positive and Negative Affect Schedule – Direction (PANAS-D) questionnaire – which can be used in order to assess the relationships between intensity and direction of affects and variables such as coping, attainment of achievement, goals and sport satisfaction – translated from French into Arabic-Tunisian language (Table 1) and administered to a cluster of athletes whose characteristics are reported in Table 2. Different factor models were tested and fitting indexes were computed to find the best solution (Table 3), whose descriptive statistics and standardized factor loadings are shown in Table 4, Table 5, respectively. The impact of gender, age and experience level are shown in Table 6.

## 2 Experimental design, materials and methods

The PANAS-D questionnaire in its original version comprises two scales (intensity and direction), each one of 20-item adjective checklist subscales (10 items corresponding to positive emotions and 10 items corresponding to negative emotions). It was translated from the French version [1] into Arabic-Tunisian, following the linguistic validation method proposed by Vallerand, termed as double translation/back translation [2].

The project received ethical approval from the Tunis University, Tunisia, and all participants provided written informed consent. Prior to data collection, permission was obtained from the team manager and the coach to conduct the study survey in athletes. Athletes were informed about the purpose and procedures of the study, and were told that the results would be made available to them upon completion of the study. Athletes who agreed to participate in the study were instructed about the survey procedures for the study. All participating athletes completed the demographic information.

A semi-confirmatory factor analysis was carried out using Factor software (version 9.2).

The multivariate analysis was performed in order to investigate the impact of parameters, such as age, gender and experience level, using SPSS (version 23.0, IBM Inc., USA).

## Figures and Tables

**Table 1:** Translated version of the Positive and Negative Affect Schedule – Direction (PANAS-D) questionnaire from French into Arabic-Tunisian language.

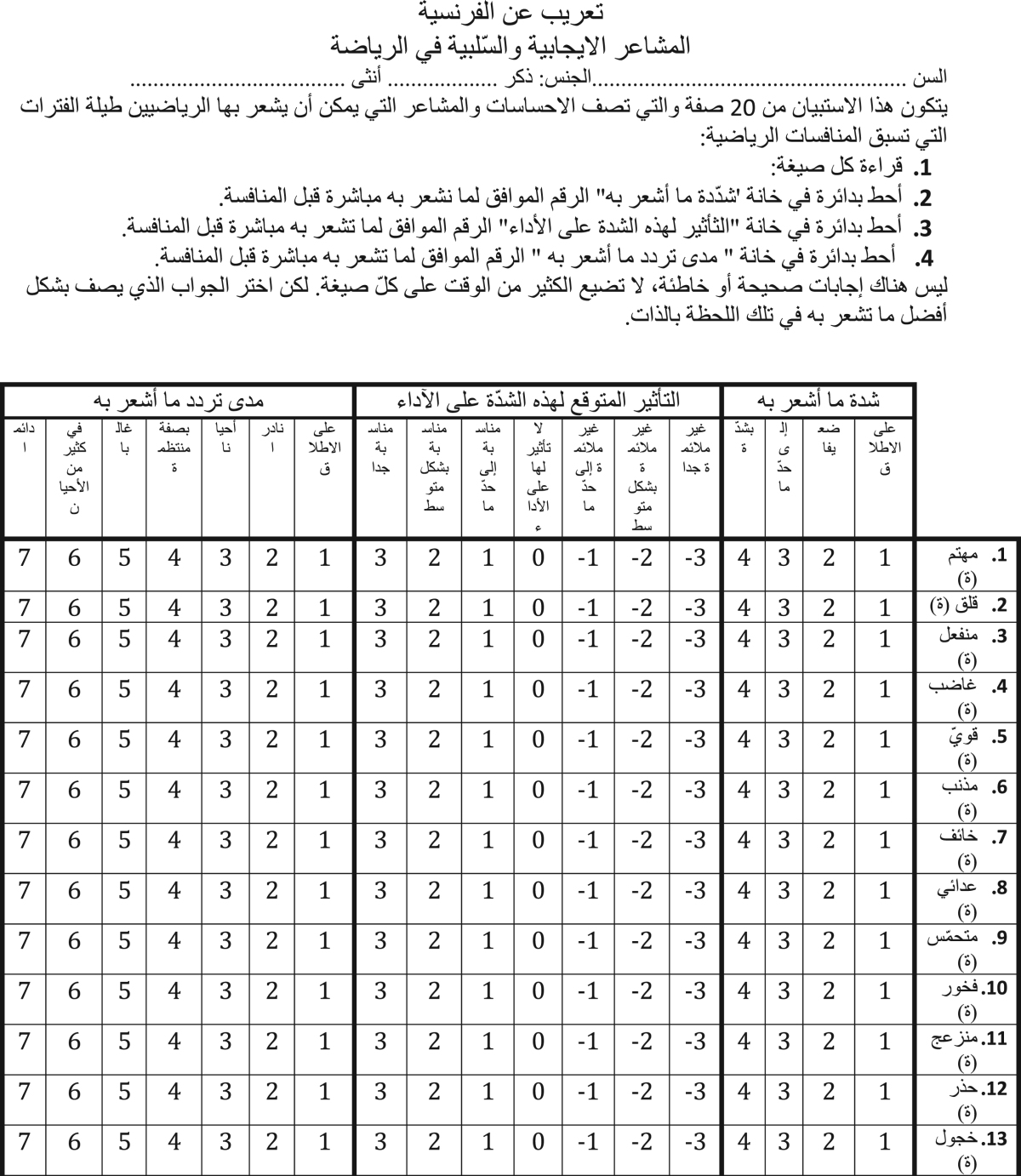
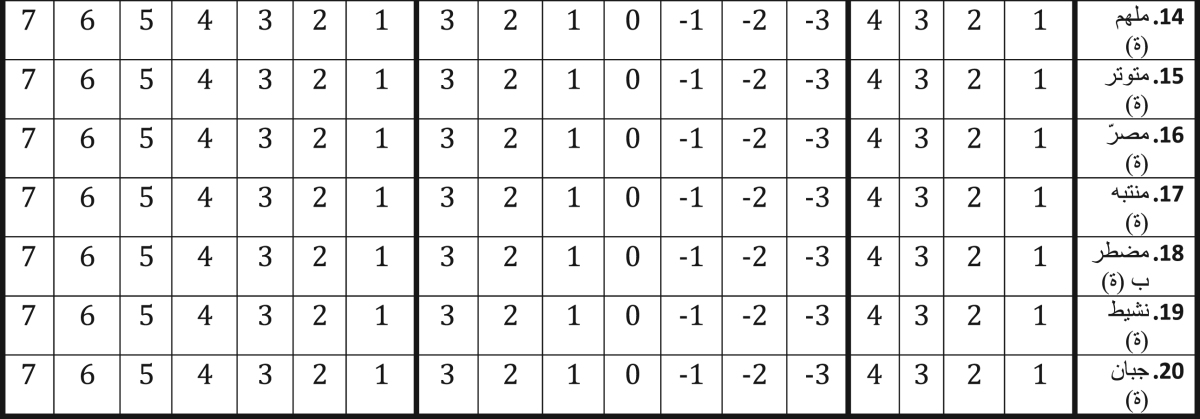

**Table 2:** General descriptive statistics of the recruited sample of athletes to which the Positive and Negative Affect Schedule – Direction (PANAS-D) questionnaire in Arabic-Tunisian language has been administered.

**Parameter**		**Value**
Age		19.43±3.78
Gender	Male	289 (55.7%)
	Female	230 (44.3%)
Experience level	International	75 (14.5%)
	National	287 (55.3%)
	Regional	130 (25.0%)
	Local	27 (5.2%)

**Table 3:** Different factor models of the Positive and Negative Affect Schedule – Direction (PANAS-D) questionnaire in Arabic-Tunisian version have been tested. Abbreviations: AGFI (adjusted goodness of fit index); CFI (comparative fit index); df (degrees of freedom); GFI (goodness of fit index); NCP (non-centrality parameter); NNFI (non-normed fit index); RMSEA (root mean square error of approximation); SRMR (standardized root mean square residual).

**Fitting index**	**Intensity**		**Direction**		**Intensity**		**Direction**		**Intensity**		**Direction**	
	**1-factor**	**1-factor (modified scale)**	**1-factor**	**1-factor (modified scale)**	**2-factor**	**2-factor (modified scale)**	**2-factor**	**2-factor (modified scale)**	**3-factor**	**3-factor (modified scale)**	**3-factor**	**3-factor (modified scale)**
χ^2^	1031.958	94.544	1469.127	223.607	670.976	30.859	754.357	47.553	369.907	192.042	546.291	303.947
Df	170	35	170	44	151	19	151	19	133	75	133	75
p-value	P=0.000010	P=0.000010	P=0.000010	P=0.000010	P=0.000010	P=0.044335	P=0.000010	P=0.000335	P=0.000010	P=0.000010	P=0.000010	P=0.000010
χ^2^ for independence model	1997.355	975.620	2960.519	1630.449	1997.355	511.509	2960.519	859.787	1997.355	1321.845	2960.519	2105.890
Df	190	45	190	55	190	36	190	36	190	120	190	120
RMSEA	0.099	0.057	0.121	0.089	0.082	0.035	0.087	0.054	0.059	0.055	0.077	0.077
NCP	220.150	45.325	220.150	56.980	195.545	24.605	195.545	24.605	172.235	97.125	172.235	97.125
NNFI	0.47	0.92	0.48	0.86	0.64	0.95	0.73	0.93	0.81	0.84	0.79	0.82
CFI	0.52	0.94	0.53	0.89	0.71	0.98	0.79	0.97	0.87	0.90	0.85	0.88
GFI	0.87	0.99	0.84	0.99	0.94	0.99	0.96	0.99	0.98	0.98	0.98	0.98
AGFI	0.86	0.99	0.82	0.98	0.92	0.99	0.95	0.99	0.97	0.97	0.97	0.97
GFI without diagonal values	0.67	0.97	0.68	0.97	0.84	0.97	0.93	0.98	0.94	0.95	0.96	0.96
AGFI without diagonal values	0.63	0.97	0.65	0.97	0.80	0.95	0.91	0.97	0.91	0.91	0.94	0.94
SRMR	0.1032	0.97	0.1311	0.0573	0.0715	0.0309	0.0622	0.0315	0.0451	0.0419	0.0495	0.0453

**Table 4:** Descriptive statistics of the best fitting 2-factor structure of the Positive and Negative Affect Schedule – Direction (PANAS-D) questionnaire in Arabic-Tunisian language. Abbreviations: df (degrees of freedom); KMO (Kaiser-Meyer-Olkin).

**Descriptive statistics**	**Intensity**		**Direction**	
	**2-factor (original, non modified scale)**	**2-factor (modified scale)**	**2-factor (original, non modified scale)**	**2-factor (modified scale)**
Determinant of the matrix	0.019989530135988	0.369785741011195	0.003029814842235	0.187834259983683
Bartlett׳s statistic	1997.4 (df=190; P=0.000010)	511.5 (df=36; P=0.000010)	2960.5 (df=190; P=0.000010)	859.8 (df=36; P=0.000010)
KMO test	0.76600	0.68598	0.82458	0.75729

**Table 5:** Standardized factor loading for intensity and direction scales of the Positive and Negative Affect Schedule – Direction (PANAS-D) questionnaire in Arabic-Tunisian language.

**Variable**	**INTENSITY**		**DIRECTION**	
	**F1**	**F2**	**F1**	**F2**
V5	0.564		0.705	
V7		0.790		0.592
V12	0.546		0.692	
V13		0.353		0.553
V15		0.559		0.652
V16	0.545		0.595	
V17	0.508		0.587	
V18		0.317		0.531
V19	0.459		0.514	
Variance	1.398	1.149	1.940	1.346
Reliability estimate	0.659	0.691	0.769	0.679

**Table 6:** Multivariate analysis of the best fitting 2-factor solution of the Positive and Negative Affect Schedule – Direction (PANAS-D) questionnaire in Arabic-Tunisian language.

**Variable**		**Value**	**F**	**Sig.**	**η**^**2**^	**Observed power**
Intercept	Pillai׳s Trace	0.714	29.385	0.000	0.714	1.000
	Wilks’ Lambda	0.286	29.385	0.000	0.714	1.000
	Lawley-Hotelling Trace	2.501	29.385	0.000	0.714	1.000
	Roy׳s Largest Root	2.501	29.385	0.000	0.714	1.000
						
Age	Pillai׳s Trace	0.158	2.208	0.000	0.158	1.000
	Wilks’ Lambda	0.842	2.208	0.000	0.158	1.000
	Lawley-Hotelling Trace	0.188	2.208	0.000	0.158	1.000
	Roy׳s Largest Root	0.188	2.208	0.000	0.158	1.000
						
Gender	Pillai׳s Trace	0.191	2.778	0.000	0.191	1.000
	Wilks’ Lambda	0.809	2.778	0.000	0.191	1.000
	Lawley-Hotelling Trace	0.236	2.778	0.000	0.191	1.000
	Roy׳s Largest Root	0.236	2.778	0.000	0.191	1.000
						
Experience level	Pillai׳s Trace	0.639	3.195	0.000	0.213	1.000
	Wilks’ Lambda	0.478	3.280	0.000	0.218	1.000
	Lawley-Hotelling Trace	0.862	3.367	0.000	0.223	1.000
	Roy׳s Largest Root	0.491	5.795	0.000	0.329	1.000
						
Gender * experience level	Pillai׳s Trace	0.513	2.434	0.000	0.171	1.000
	Wilks’ Lambda	0.562	2.489	0.000	0.175	1.000
	Lawley-Hotelling Trace	0.651	2.544	0.000	0.178	1.000
	Roy׳s Largest Root	0.377	4.449	0.000	0.274	1.000
